# Transcription Factor HusR (YnfL) Is a Novel Regulator for Hydroxyurea Sensitivity in *Escherichia coli* K-12

**DOI:** 10.3390/microorganisms14010134

**Published:** 2026-01-07

**Authors:** Ikki Kobayashi, Sousuke Imamura, Ken Okamoto, Tomohiro Shimada

**Affiliations:** 1 School of Agriculture, Meiji University, 1-1-1, Kawasaki-shi 214-8571, Kanagawa, Japan; 2 Space Environment and Energy Laboratories, NTT, Inc., Musashino-shi 180-8585, Tokyo, Japan; sousuke.imamura@ntt.com (S.I.); ken.okamoto@ntt.com (K.O.)

**Keywords:** transcription factor, hydroxyurea sensitivity, DNA damage, *Escherichia coli*

## Abstract

Understanding the functions of all genes and their biological mechanisms based on comprehensive genome regulation mechanisms is an important issue in life sciences. YnfL is an uncharacterized LysR family transcription factor in *Escherichia coli*. Genomic SELEX screening was performed to identify YnfL regulatory targets in the *E. coli* genome and reveal the function of YnfL. Nine loci on the *E. coli* genome were identified as regulatory targets of YnfL, and the target genes were involved in supplying DNA substrates and DNA repair. RT-qPCR analysis in vivo revealed that YnfL activates its target genes during the stationary phase. Tests for drug resistance that causes DNA damage revealed that *ynfL* deficiency increased abnormal cell filamentation and the appearance of anucleate cells in the presence of hydroxyurea. Furthermore, *ynfL* deficiency reduced cell survival under long-term nitrogen starvation conditions. In summary, we propose renaming *ynfL* to hydroxyurea sensitivity regulator (*husR*). These findings contribute to understanding DNA maintenance and long-term survival through transcriptional regulation.

## 1. Introduction

Genomic stability is constantly threatened by a wide range of damaged nucleotides arising from endogenous and exogenous genotoxic stresses. Therefore, when cells suffer DNA damage, they repair it using their own DNA repair system [1,2], such as homologous recombination [3]. In addition, DNA repair and maintenance require continuous energy consumption and dissipation at of large levels [4]. However, situations in which cellular energy reserves are severely depleted or complete template is unavailable are common. This is particularly pronounced under stress conditions such as prolonged starvation or continuous exposure to DNA-damaging agents [5]. For example, ribonucleoside diphosphate reductase and the dNTP pool are involved in maintaining the genome stability of living cells [6]. Filamentation has been observed in the model microorganism *Escherichia coli* (*E. coli*) in response to physiological DNA damage and to DNA damage induced by UV radiation or antibiotic treatment [7]. Although the cellular functions and physiological effects of DNA damage are well understood, the regulatory mechanisms governing the responses to such stress remain poorly understood.

*E. coli*, a model microorganism, contains approximately 4500 protein-coding sequences in its genome [8,9] and alters the expression patterns of these genes in response to environmental changes and stress [10,11]. The genes required for responses to various stresses and survival have been identified through comprehensive analyses using gene-deletion or overexpression strains [12,13]. At present, even in the model bacterium *E. coli*, the functions of about one-quarter of its genes remain unknown, largely because many stress-response genes are not expressed under standard laboratory conditions. Accordingly, a full set of transcription factors (TFs) involved in the expression of stress conditions has not yet been identified. When the TFs and conditions that influence the expression of regulatory functions are unknown, conventional in vivo approaches are ineffective for identifying the regulatory targets of previously uncharacterized TFs, since these TFs are not always expressed or functional [14]. At present, the function of one-fifth of *E. coli* TFs remains unknown [15,16].

To rapidly identify the regulatory targets of uncharacterized TFs, we developed an improved system of genomic systematic evolution of ligands using exponential enrichment (gSELEX) [17,18] and successfully identified regulatory targets for several TFs [15]. This gSELEX screening system, which relies on in vitro interactions between purified test TF proteins and genomic DNA fragments, is particularly effective for identifying regulatory targets of previously uncharacterized TFs when their expression conditions are unknown. Using the gSELEX system, we identified the regulation targets and regulatory roles for some unknown Y-TFs, including YagI (renamed to XynR), YbaO (renamed to DecR), YbiH (renamed to CecR), YbjK (renamed to RcdA), YcdC (renamed to RutR), YcjZ (renamed to PgrR), YdeO (renamed to PhhR), YdcN (renamed to SutR), YdfH (renamed to RspR), YdhM (renamed to NemR), YeaM (renamed to NimR), YedW (renamed to HprR), YgfI (renamed to SrsR), YgiP (renamed to Dan), YiaJ (renamed to PlaR), YiaU (renamed to CsuR), and YihW (renamed to CsqR) [18]. gSELEX analysis can also overcome the difficulties of in vivo analysis because a set of regulatory proteins that regulate a single promoter compete to bind to overlapping DNA sites [19,20]. Bacterial DNA-binding TFs regulate nearby genes by binding to DNA; therefore, the regulation of target promoters, genes, and operons can be predicted from the locations of test TF recognition sequences.

The research purpose of this study was to understand the function of a comprehensive set of *E. coli* TFs, and we sought to characterize YnfL, which has been recognized as an uncharacterized TF in *E. coli* and belongs to the LysR family [15]. A systematic search was conducted using the gSELEX-chip system to identify the target genes regulated by YnfL [18]. A total of nine YnfL-binding sites were identified throughout the *E. coli* genome. Predicted YnfL target genes are involved in supplying DNA substrates and DNA repair. The transcriptional regulatory and physiological roles of YnfL were examined experimentally. As a result, YnfL determines the sensitivity to hydroxyurea, which inhibits ribonucleoside diphosphate reductase for dNTP synthesis. Therefore, we propose renaming YnfL to HusR (a hydroxyurea sensitivity regulator). The physiological significance of HusR was discussed.

## 2. Materials and Methods

### 2.1. Bacterial Strains and Plasmids

The *Escherichia coli* K-12 W3110 type A [21] genome was used to construct the DNA library required for gSELEX screening. *E. coli* BW25113 [22] and its single-gene knockout mutant JW1587 for *husR* [23] were obtained from the *E. coli* Stock Center (National Bio-Resource Center, Chiba, Japan). Cells were grown in an LB medium (10 g L^−1^ Tryptone, 10 g L^−1^ Sodium Chloride, and 5 g L^−1^ Yeast Extract) or M9 minimal medium (42 mM Na_2_HPO_4_, 22 mM KH_2_PO_4_, 8.5 mM NaCl, 18.5 mM NH_4_Cl, 2 mM MgSO_4_, and 0.1 mM CaCl_2_) containing 0.2% glucose and 0.2% casamino acids, at 37 °C with constant shaking at 150 rpm. When required, 20 μg mL^−1^ kanamycin was added to the medium. Cell growth was monitored by assessing optical density at 600 nm (O.D._600_) using ODBox-C (TAITEC, Saitama, Japan).

To construct the *husR* promoter-fused GFPuv plasmid, a vector fragment containing the ColE1 ori, ampicillin resistance gene, and the GFPuv gene was amplified by PCR from the pGFPuv vector (Takara Bio, Shiga, Japan) using the primers GFPuv-F/GFPuv-R [Table S1C]. The DNA fragment containing the promoter region of *husR* was amplified using the primers PhusR-F/PhusR-R, with BW25113 genomic DNA as the template. These fragments were joined by Gibson assembly [24], and the resulting plasmid was designated pPhusR-GFPuv.

### 2.2. Purification of HusR Protein

The pHusR plasmid for expressing and purifying HusR was constructed according to standard procedures [25]. In brief, HusR coding sequences were PCR-purified using *E. coli* K-12 W3110 genomic DNA as a template and inserted into the pET21a (+) vector (Novagen, Darmstadt, Germany) between the NdeI and NotI sites, yielding pHusR. The pHusR expression plasmid was transformed into *E. coli* BL21 (DE3) cells. Transformants were grown in an LB medium, and HusR was expressed by adding IPTG to the middle of the exponential phase. HusR was purified by affinity purification using a Ni-nitrilotriacetic acid agarose column. The affinity-purified HusR protein was frozen in storage buffer at −80 °C until use. Protein purity was greater than 95% as determined by sodium dodecyl sulfate-polyacrylamide gel electrophoresis.

### 2.3. Genomic SELEX (gSELEX) Screening of HusR-Binding Sequences

gSELEX screening was performed as previously described [17,18]. Briefly, DNA fragments from the *E. coli* K-12 W3110 genome were generated by sonication of purified genomic DNA and subsequently cloned into the multicopy plasmid pBR322. For each gSELEX screen, the DNA mixture was regenerated using PCR. For gSELEX screening, 5 pmol of DNA fragments and 10 pmol of HusR were mixed in binding buffer (10 mM Tris-HCl, pH 7.8 at 4 °C, 3 mM magnesium acetate, 150 mM NaCl, and 1.25 mg mL^−1^ bovine serum albumin). The SELEX cycle was repeated four times to enrich sequences that bind HusR. SELEX fragments were mapped across the *E. coli* genome using a gSELEX-chip system with a 43,450-feature DNA microarray [26]. The genomic SELEX sample obtained with HusR was labeled with Cy3, while the original genomic DNA library was labeled with Cy5. After hybridization to the DNA tiling array (Agilent Technologies, Santa Clara, CA, USA), the Cy3/Cy5 ratios were measured, and peak signals were plotted according to the positions of DNA probes along the *E. coli* K-12 genome.

### 2.4. Gel Shift Assay

The gel shift assay was performed according to the standard procedure [27]. Probes for HusR-binding target sequences were generated by PCR amplification using a pair of primers (Table S1A) and Ex Taq DNA polymerase (Takara Bio, San Jose, CA, USA). A mixture of each probe and HusR was incubated at 37 °C for 30 min in the binding buffer. After addition of the DNA-loading solution, the samples were directly subjected to PAGE. The DNA in the gels was stained with GelRed (Biotium, Fremont, CA, USA) and detected using LuminoGraph I (Atto, Amherst, NY, USA).

### 2.5. RT-qPCR Analysis

RT-qPCR was performed according to standard procedures [28]. *E. coli* cells were inoculated into M9 minimal medium supplemented with 0.2% glucose and 0.2% casamino acids an at 37 °C under aeration, with constant shaking at 150 rpm. After inoculation, the total RNA was extracted using ISOGEN solution (Nippon Gene, Tokyo, Japan). Total RNA was transcribed to cDNA with random primers using the THUNDERBIRD SYBR qPCR RT Set (TOYOBO, Osaka, Japan). Quantitative PCR (qPCR) was conducted using the THUNDERBIRD SYBR qPCR Mix (TOYOBO) and a LightCycler 96 system (Roche, San Francisco, CA, USA). The primer pairs are listed in Supplementary Table S1B. The cDNA templates were serially diluted four-fold and used for qPCR. Each qPCR reaction contained 10 μL of THUNDERBIRD SYBR qPCR Mix (TOYOBO), 1 μL of each primer (6 μM stock), 7 μL of water, and 1 μL of cDNA. Amplification was performed with an initial denaturation at 95 °C for 2 min, followed by 45 cycles of 10 s at 95 °C and 20 s at 55 °C, and then incubation for 20 s at 72 °C. Expression levels were normalized to 16S rRNA, and relative expression was calculated using Roche Relative Quantification software (LightCycler^®^96 Version 1.1.0.1320). The results are presented as the average of three independent experiments.

### 2.6. Drug Sensitivity Tests

*E. coli* cells were grown by shaking at 150 rpm, at 37 °C in M9 minimal medium containing 0.2% glucose and 0.2% casamino acid after a 1:100 dilution of overnight culture. Growth was monitored by measuring the absorbance at 600 nm. Antibiotics were used at the following concentrations: 50, 100, and 200 mM hydroxyurea; 5, 10, and 25 μg mL^−1^ nalidixic acid; 5, 10, and 25 μg mL^−1^ acriflavine; and 5, 10, and 25 μg mL^−1^ rifampicin. To measure the viable cell count, cells were diluted and spread onto LB agar plates to count colony forming units. After incubating the plates at 37 °C, the number of colonies were counted. All the results are presented as the average of three independent experiments.

### 2.7. UV Sensitivity Test

*E. coli* cells were inoculated into M9 minimal medium containing 0.2% glucose and 0.2% casamino acid, cultured for 24 h under aerobic conditions with constant shaking at 150 rpm, diluted, and spread onto LB agar plates (LB medium containing 15 g L^−1^ Agar). Subsequently, the plates were irradiated with UV light at 0, 4, 8, and 12 mJ/cm^2^ using a UVP Crosslinker (Analytik Jena, Jena, Germany). After incubating the plates at 37 °C overnight, the number of colonies on the plates was counted. The survival rate was calculated as the ratio of the colony counts of the UV-irradiated and non-irradiated cells. The results are presented as the average of three independent experiments.

### 2.8. Microscopy

*E. coli* cells were grown in M9 minimal medium containing 0.2% glucose and 0.2% casamino acid for 24 h with 0, 50, or 100 mM hydroxyurea. After cultivation, 1 mL of the cells was pelleted and resuspended in 0.1 mL of Phosphate-Buffered Saline containing 1.0% glutaraldehyde. The cells were then pelleted and resuspended in 10 μg mL^−1^ Hoescht 33342 solution, and incubated at room temperature for 20 min. Images were captured using a KEYENCE fluorescence microscope (BZ-800L/BZ-810, KEYENCE, Osaka, Japan). Hoechst fluorescence images were observed under a fluorescence microscope using a filter unit OP-87762 (KEYENCE; excitation: 360/40 nm, emission: 460/50 nm, dichroic mirror: 400 nm).

### 2.9. Survivability Test Under Nitrogen Depletion

*E. coli* cells were grown overnight in M9 minimal medium containing 0.2% glucose and 0.2% casamino acid at 37 °C and 150 rpm. The cells were cultured overnight, and then they were transferred into 3 mM or 20 mM NH_4_Cl Gutnick minimal medium [29] (5.74 mM K_2_SO_4_, 77.5 mM K_2_HPO_4_, 33.8 mM KH_2_PO_4_, 0.41 mM MgSO_4_, 0.4% Glucose), using NH_4_Cl as the sole nitrogen source, and grown at 37 °C and 150 rpm. After the indicated times, the cells were diluted and spread onto LB agar plates. The number of colonies was counted using a Scan300 colony counter (Interscience, Saint Nom, France) after incubating the plates at 37 °C overnight. The results are presented as the average of three independent experiments.

### 2.10. Statistical Analysis

Data obtained were reported as the mean ± standard deviation unless otherwise indicated. Statistically significant differences were assessed by using Student’s *t*-test with a two-tailed test. Statistical significance was reported as follows: ** *p* < 0.01; * *p* < 0.05.

## 3. Results

### 3.1. Regulation Targets of HusR by gSELEX-Chip Screening

To identify the set of HusR-binding sequences, we performed gSELEX screening [18]. Briefly, purified His-tagged HusR was incubated with a pool of *E. coli* genomic DNA fragments 200–300 bp in length, and the HusR-bound fragments were isolated by affinity purification. The original genomic DNA fragments formed smeared bands on PAGE; however, DNA fragments with a high affinity for HusR were enriched after four cycles of gSELEX, forming sharper bands on PAGE gels.

**Figure 1:**
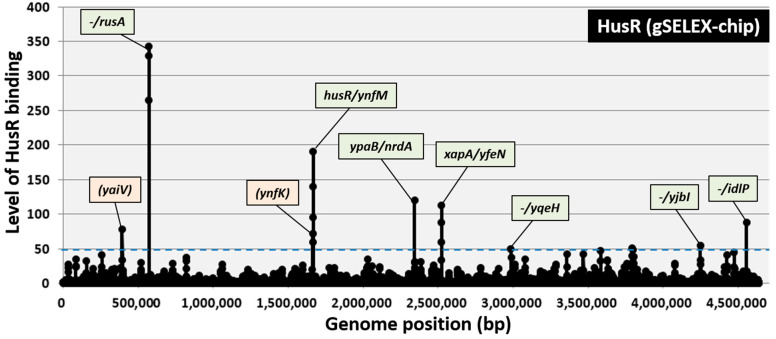
Identification of HusR-binding sites on the *E. coli* K-12 genome by gSELEX-chip. gSELEX screening of DNA-binding sequences was performed for HusR (renamed YnfL), a yet uncharacterized LysR-family TF of *E. coli*, using purified C-terminal His-tagged HusR and a library of DNA segments from the *E. coli* K-12 W3110 genome. Following gSELEX, a collection of DNA fragments was subjected to gSELEX-chip analysis using the tiling array of the *E. coli* K-12 genome. The cut-off level of 50 is shown by a blue dotted line, and the list of all HusR-binding sites from setting this cut-off level is given in Table 1. Peaks shown in green represent the HusR-binding sites inside spacer regions, whereas peaks shown in orange represent the HusR-binding sites inside ORFs.

gSELEX fragments were labeled with Cy3, while the original genomic DNA library was labeled with Cy5 to identify the comprehensive set of binding sites for HusR. A mixture of fluorescently labeled samples was hybridized to an *E. coli* DNA-tiling microarray [30]. The fluorescence intensity ratio for each probe, comparing the HusR-bound sample with the original DNA library, was measured and plotted along the *E. coli* K-12 genome. The signal strength reflected the binding affinity of HusR. After setting the cutoff level to 50, nine peaks were identified in the gSELEX-chip pattern, comprising seven intergenic regions and two intragenic regions (Figure 1). The highest peak was observed in the region upstream of the *rusA* gene. Based on the criterion that prokaryotic TF-binding sites are located upstream of their regulatory target genes [19,31], ten genes or operons (*rusA-ylcG*, *husR*, *yhfM*, *ypaB*, *nrdAB*, *xapA*, *yfeN*, *yqeH*, *yjbI*, and *idlP-iraD*) were predicted to be potential regulatory targets of HusR (Table 1). The identification of the HusR-binding site within its own promoter region suggests that the HusR regulon is controlled by HusR through autogenous regulation.

### 3.2. Confirmation of the Predicted HusR-Binding to the Target Promoters

**Figure 2:**
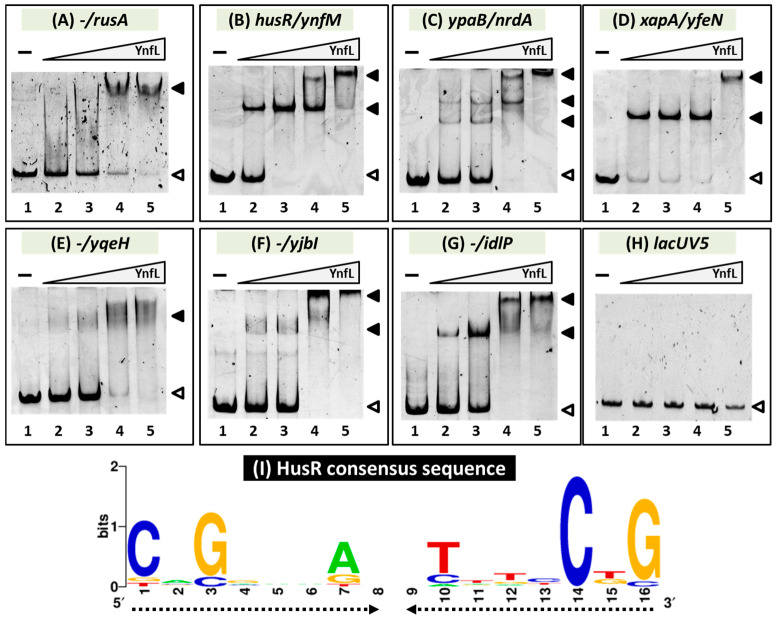
Confirmation of HusR binding in vitro to the regulatory promoters. (**A**–**H**) Purified HusR was mixed with 0.2 pmol of each target DNA probe to the HusR-binding regions shown in Figure 1. HusR added were (in pmol): lane 1, 0; lane 2, 7.5; lane 3, 15; lane 4, 30; and lane 5, 60. Filled triangles indicate the HusR–DNA probe complex, whereas open triangles indicate free probes. (**I**) Palindromic consensus sequence of HusR-box. Sequences of the probes with HusR-binding activity were analyzed using the MEME program (https://meme-suite.org/meme/ (accessed on 10 November 2025)) (see Table 1). WEBLOGO (http://weblogo.berkeley.edu/logo.cgi (accessed on 10 November 2025)) was used for matrix construction. The proportional height of each individual character represents the conservation of the corresponding nucleotide.

We performed a gel shift assay to detect HusR target DNA complexes to confirm the in vitro binding of HusR to the target sites predicted based on the gSELEX pattern. DNA probes were prepared for all the targets and mixed with increasing amounts of purified HusR. All seven DNA probes formed HusR concentration-dependent HusR–DNA complexes [Figure 2A–G]. In addition, multiple HusR-DNA complexes were observed for several probes, suggesting that these promoters possessed multiple HusR-binding sites [Figure 2B–D,F,G]. In contrast, binding was not observed in the nonspecific *lacUV5* promoter region, which was used as a negative control [Figure 2H]. These results indicate the specific binding of HusR to these seven sites and agree with their detection by gSELEX screening.

A collection of 500 bp sequences from the HusR targets was analyzed using the MEME program [32] to identify the HusR recognition sequence. A palindromic 16 bp long sequence [Figure 2I] was found in all HusR-bound regions [Table 1]. Multiple sequences were detected in most of the regions. This was consistent with the observation of multiple HusR-DNA probe complexes in gel shift assays. Based on this, a 16 bp palindromic sequence was suggested as the consensus sequence for HusR binding.

### 3.3. Regulatory Role of HusR in the Expression of the Target Genes In Vivo

**Figure 3:**
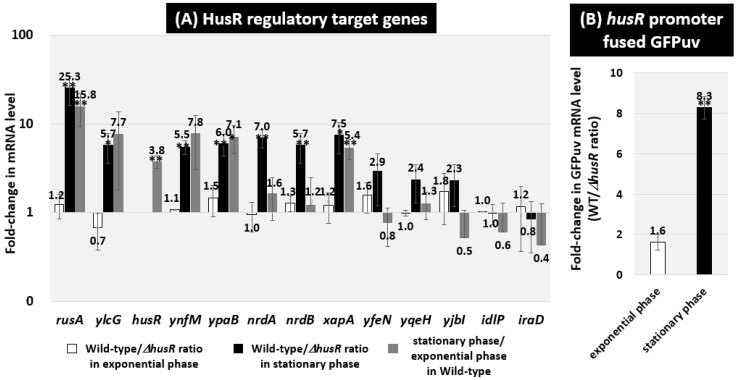
In vivo influence of *husR* deletion on transcription level of the HusR targets by RT-qPCR. *E. coli* wild-type BW25113 and its *husR* deletion mutant JW1587 were grown in the M9 minimal medium supplemented with 0.2% glucose and 0.2% casamino acids at 37 °C. Total RNA was prepared from each strain at 3 h (in exponential phase; O.D._600_ = 0.5) or 24 h (in stationary phase; O.D._600_ = 2.0) cultured cells and subjected to RT-qPCR analysis. The y-axis represents the relative level of mRNA of each HusR target gene between the wild-type and the *husR* mutant in the exponential (white bar) or in the stationary (black bar) phases, as well as the relative level between the wild-type in exponential phase and the stationary phase (gray bar) (**A**). *E. coli* wild-type BW25113 pPhusR-GFPuv and its *husR*-deleted mutant JW1587 harboring pPhusR-GFPuv were performed in the same manner, and compared in exponential phase (white bar) and stationary phase (black bar), respectively (**B**). The ratio of 16S rRNA was set as an internal control between the compared strains. Each experiment was repeated three times, and the mean ± SD are shown. Statistically significant differences were also determined by Student’s *t*-test with a two-tailed test and are indicated with lines and asterisks (** *p* < 0.01, * *p* < 0.05).

We used RT-qPCR to measure the mRNA levels in vivo for each predicted HusR target genes in the *E. coli* wild-type strain and *husR* deletion strain to assess the potential effect of HusR on the target promoters identified in vitro based on HusR-binding sites [Figure 3A]. Total RNA was prepared from each strain—grown in LB medium—and the mRNA levels of the individual HusR target genes were measured by RT-qPCR using total RNA prepared from both exponential and stationary growth phases Comparing mRNA levels between the wild-type strain and *husR* deletion strain, no significant differences were observed for any target genes in exponential growth phase cells [Figure 3A, white bar]. In contrast, the wild-type strain showed a more than five-fold increase in the ratios of *rusA-ylcG*, *ynfM*, *ypaB*, *nrdAB*, and *xapA* in the stationary phase [Figure 3A, black bar]. When comparing the mRNA levels during the exponential and stationary phases in the wild-type strain, a similar increase was observed in the stationary phase for targets other than *nrdAB* [Figure 3A, gray bar]. These results suggest that HusR acts as an activator of these target genes and that they are activated in a HusR-dependent manner during the stationary phase. For *nrdAB*, it was suggested that the expression level of *nrdAB* decreased during the stationary phase in the*husR*-deficient strain. In the wild-type strain, the expression level of *husR* mRNA itself was also increased during the stationary phase. Therefore, to measure the activity of the *husR* promoter using the reporter GFPuv gene, we constructed a plasmid containing a fusion cassette of the *husR* promoter and GFPuv, designated pPhusR-GFPuv. Initial attempts to measure GFP in cells harboring pPhusR-GFPuv were unsuccessful because of low fluorescence intensity. Therefore, we measured the GFPuv mRNA levels using RT-qPCR and compared wild-type and *husR*-deficient strains. Similarly to the HusR-regulated genes, no changes were observed during the exponential phase. In contrast, an approximately eight-fold increase was observed in the stationary phase [Figure 3B], suggesting that *husR* is activated by autoregulation. Therefore, we propose that HusR functions as an activator of its regulatory target genes, including *husR* itself, during the stationary phase.

### 3.4. Role of HusR in Resistance to DNA Damage

The functions of HusR target genes are classified as supplying DNA substrates, including *nrdAB* (ribonucleoside-diphosphate reductase for the dNDP de novo biosynthesis) and *xapA* (xanthosine phosphorylase for purine salvage and degradation), and being involved in DNA damage response, including *rusA* (endodeoxyribonuclease for homologous recombination in Holliday junction resolvase). In relation to DNA damage, mutants with partial disruption of NrdA function showed a greater number of double-strand breaks caused by replication fork stalling [33] and expression of *iraD* (an anti-adaptor protein for the inhibition of proteolysis of stationary phase sigma factor RpoS by interacting with RssB), in which the HusR target gene is induced by DNA damage in the SOS response [34,35]. Other genes have ambiguous or unknown functions, but overexpression of *ynfM* (a putative transporter) in mutants that lack the major efflux pump permease *acrB* resulted in hypersensitivity to acriflavine [36], which causes DNA damage through intercalation and inhibition of DNA repair. In addition, *yjbI* (a functionally unknown conserved protein) suppresses *dnaG* DNA primase during DNA synthesis by DNA polymerase III under DNA damage conditions [37]. Thus, the functions of the genes in the HusR regulon suggest that HusR plays a role in DNA synthesis and the DNA damage response.

**Figure 4:**
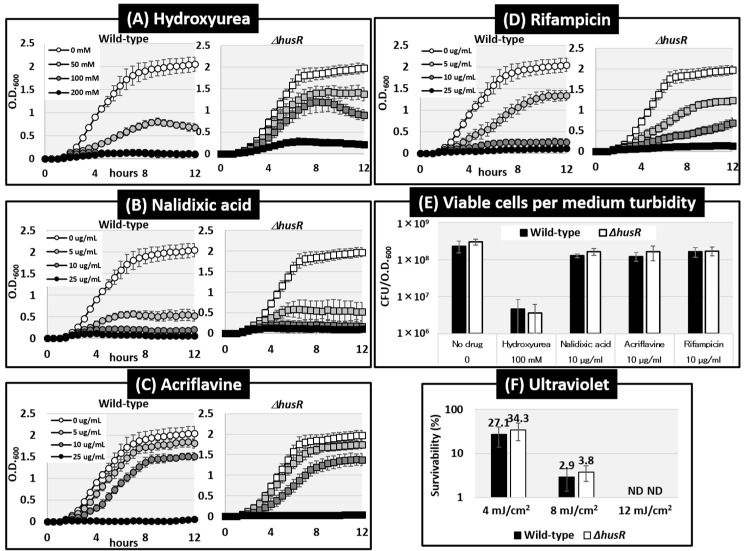
Influence of *husR* deletion on effects of DNA-damage inducers on growth curves or survivability. Growth curves of *E. coli* wild-type BW25113 and its *husR*-deleted mutant JW1587 were measured by O.D._600_ every 30 min in the presence of Hydroxyurea (**A**), Nalidixic acid (**B**), Acriflavine (**C**), and Rifampicin (**D**) at the concentrations indicated on the slide, respectively. The number of viable cells in the culture medium turbidity at the 12 h time point under the presence of specific concentrations of compounds from (**A**) to (**D**) was evaluated by measuring colony-forming units (CFU) relative to optical density (O.D._600_) (**E**). The compounds and concentrations tested were: no drug (Control), 100 mM hydroxyurea, 10 μg mL^−1^ nalidixic acid, 10 μg mL^−1^ acriflavine, and 10 μg mL^−1^ rifampicin. The survival rate following UV irradiation was calculated by measuring and comparing the number of colony forming units between irradiation and non-irradiation conditions (**F**). Error bars indicate standard deviations of results from at least three independent experiments. ND means Not Detected.

We examined the effects of *husR* deletion on compounds that cause DNA damage or inhibit replication. For this purpose, we measured the growth curves of wild-type and *husR* mutant strains in the presence of four drugs with different mechanisms of action: hydroxyurea (HU), nalidixic acid, acriflavine, and rifampicin, at different concentrations [Figure 4A–D]. Increased concentrations of all the drugs inhibited *E. coli* growth. Regarding HU, the turbidity after 12 h of cultivation in wild-type and *husR*-deficient strains was O.D._600_ = 0.70 and 1.37 at 50 mM, and 0.11 and 0.89 at 100 mM, indicating an increase in the turbidity due to the deletion of *husR*. At certain HU concentrations, a slight decrease in turbidity was observed after transition into stationary phase, suggesting that some abnormality may have occurred in the cell morphology. No other significant effects were observed for the test inhibitors under these conditions. To further evaluating the effects of the drugs based on the medium turbidity, the viable cell count was evaluated. Measurement of viable cell counts relative to the medium turbidity in certain drug concentrations revealed that *E. coli* in the presence of HU exhibited a significantly lower viable cell counts relative to the medium turbidity compared to other drugs, being tens of times lower [Figure 4E]. These results suggest that the deficiency of *husR* in the presence of HU does not significantly affect viable cell counts but does affect medium turbidity, implying an impact on cell proliferation and elongation. Simultaneously, this growth-inhibitory effect of HU, unlike that of other drugs, does not correlate with simple viable cell counts. Note that the survival rates were measured after exposure to different ultraviolet intensities; however, no effect of *husR* deficiency was observed [Figure 4F].

### 3.5. Physiological Role of HusR Under Hydroxyurea Pressure

**Figure 5:**
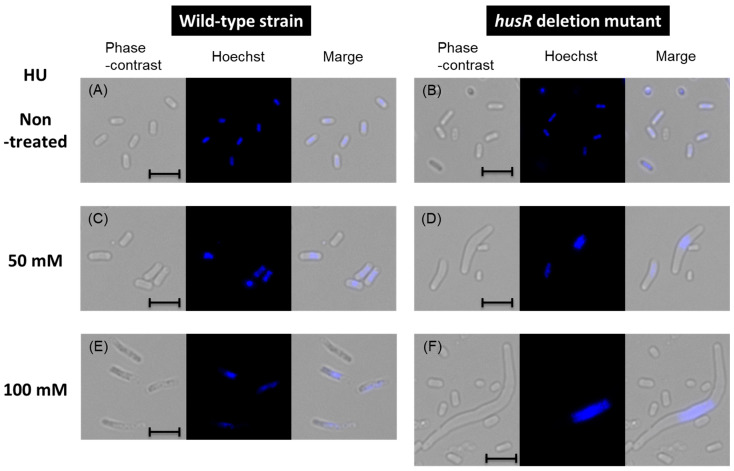
Influence of *husR* deletion on cell morphology and chromosome under the presence of HU. Phase-contrast and fluorescence images of *E. coli* wild-type BW25113 (**A**,**C**,**E**) or ∆*husR* JW1587 (**B**,**D**,**F**) growing at 37 °C for 24 h without HU (**A**,**B**), with 50 mM HU (**C**,**D**), and with 100 mM HU (**E**,**F**). The scale bar indicates 5 µm.

HU is an inhibitor of nucleoside diphosphate (NDP) reductase encoded by *nrdAB*, a HusR target genes. Continuous activity of NDP reductase is essential for continuous DNA synthesis, and its inhibition immediately stops replication. Riola et al. reported that *E. coli* lacking temperature-sensitive NrdA enzyme activity exhibited abnormal cell elongation and frequently produced anucleate cells in the presence of HU [38]. Our HU resistance test (Figure 4) was evaluated for turbidity based on the absorbance at 600 nm; therefore, it was considered that abnormal cell length or abnormal cell proliferation might have been reflected. We examined the morphology and nuclear state of the *husR*-deficient strain under similar conditions. After 24 h of cultivation under different HU concentrations, wild-type and *husR* mutant strains were measured for cell size, and chromosome state was observed using a fluorescent microscope [Figure 5 and Table 2]. The cell length in the absence of HU was approximately 2 μm for both the wild-type and *husR*-deficient strains [Figure 5A,B]. Addition of 50 mM and 100 mM HU to the medium resulted in cell length elongation to 3.7 μm and 5.9 μm, respectively, in the wild-type strain [Figure 5C,E]. In the *husR* mutant strain, significant cell length elongation to 5.4 μm and 9.6 μm was observed [Figure 5D,F], those with a length of more than 15 μm accounted for 14% of the total cells, with the largest cell observed reaching approximately 70 μm [Figure S1]. Furthermore, the observation of chromosomal states revealed that in the absence of HU, both the wild-type and *husR* mutant strains retained chromosomes in all cells [Figure 5A,B]. However, in the presence of 50 mM HU, 25% of the cells in both strains were anucleated [Figure 5C,D]. At 100 mM HU, the proportion of anucleated cells was 18% in the wild-type strain and increased significantly to 74% in the *husR* mutant strain [Figure 5E,F]. Table 2 summarizes the turbidity, cell length, and percentage of anucleated cells of each strain in the presence of HU. These results indicated that when *E. coli* proliferates in the presence of HU, a certain proportion of anucleate cells appeared. In the presence of HU, the wild-type strain maintained a relatively normal cell population by suppressing cell proliferation by HusR. In contrast, deletion of *husR* suggests that remarkable cell elongation occurs and proliferation proceeds as abnormal cells in the form of anucleate cells. Taken together, we propose renaming YnfL to HusR (a regulator of hydroxyurea sensitivity).

### 3.6. The Effect of husR Deficiency on Long-Term Survival Under Nitrogen Starvation

**Figure 6:**
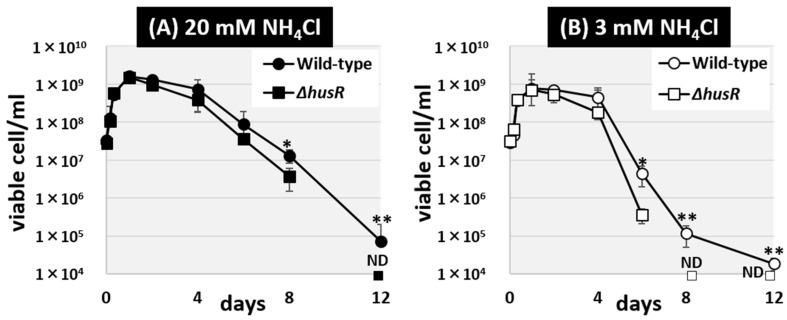
Influence of *husR* deletion on long-term survival under nitrogen starvation. Survivability in Gutnick medium supplemented with 0.4% glucose with 20 mM NH_4_Cl (**A**) or 3 mM (**B**) as the sole nitrogen source, of *E. coli* wild-type BW25113 and its *husR*-deleted mutant JW1587 was measured over days by counting the colony forming units. Each experiment was repeated three times, and the mean ± SD are shown. Statistically significant differences were also determined by Student’s *t*-test with two-tailed test and are indicated with lines and asterisks (** *p* < 0.01, * *p* < 0.05). ND means Not Detected.

The alteration in HU sensitivity due to *husR* deficiency is thought to be caused by dNTP depletion, resulting from the inhibition of NrdAB ribonucleoside-diphosphate reductase. Since the regulatory targets of HusR include genes involved in purine salvage and DNA repair, HusR is thought to be involved in DNA damage caused by DNA substrate depletion in the stationary phase. Therefore, we hypothesized that one of the fundamental roles of HusR in the natural environment is to maintain nucleic acids during the long-term survival of *E. coli*. To test this hypothesis, we tested the long-term survival of *E. coli* deprived of nitrogen, an element constituting nucleic acids. Using the Gutnick minimal medium, which is commonly employed to induce nitrogen deficiency in *E. coli*, we observed the long-term survival of wild-type and *husR*-deficient strains under both nitrogen-rich [Figure 6A] and nitrogen-poor conditions [Figure 6B]. Over a 12-day period, the number of viable *E. coli* cells in the culture medium was measured. In all culture media, proliferation occurred until the first day of culture. Subsequently, the viable cell count was maintained until approximately the fourth day, after which it began to decrease from the sixth day onward. In a nitrogen-rich medium, the survival rate of the *husR*-deficient strain decreased to one-third of that of the wild-type strain [Figure 6A]. In contrast, in nitrogen-poor medium, the survivability of the *husR*-deficient strain decreased more than tenfold by the sixth day and could no longer be detected by the eighth day [Figure 6B]. Taken together, we propose that physiological role of *husR* is ensuring long-term survival by maintaining chromosomes, particularly under nitrogen deficiency.

## 4. Discussion

Fully understanding the *E. coli* transcriptional regulatory network—including all seven sigma factors and roughly 300 TFs—and defining the direct targets of each regulatory protein remain a major challenge [15,20,39]. Transcriptional regulators recognize and bind to specific sequences in genomic DNA, thereby controlling the transcription of nearby functionally related genes. Therefore, even when the function of a transcription factor is unknown, identifying its binding sites on the genome can reveal its function and physiological role. However, analyzing transcription factors with unidentified functions is particularly difficult because the conditions under which these factors are expressed and activated in vivo remain unknown. Thus, we previously used our independently developed gSELEX method to elucidate the functions of more than 15 TFs with unknown functions by identifying genomic transcriptional regulatory networks [17,18]. Recently, the YnfL-binding site was identified in vivo using ChIP-exo, and only the intergenic region between *ynfL* and *ynfM* was identified as the binding site [40]. However, the regulation has not been confirmed via other methods. In this study, we identified nine binding sites for YnfL, including the intergenic region between *ynfL* and *ynfM*, using the gSELEX method [Figure 1 and Figure 2; Table 1]. A 16 bp sequence was suggested as the binding consensus sequence for HusR, and its palindromic sequence matched the typical characteristics of transcription factors [Figure 2I] [19]. The in vitro and in vivo analyses suggest that HusR activates several target genes, including *rusA-ylcG*, *ynfM*, *ypaB*, *nrdAB*, *xapA*, and *husR* itself, all of which are located adjacent to the HusR-binding sequence in the *E. coli* genome [Figure 3].

HusR binds to its promoter and is self-activated during the stationary phase [Figure 3]. The activation of its target genes was also observed during the stationary phase. These results are consistent with reports that HusR target genes such as *ylcG* and *iraD* were induced during the stationary phase [34,35,41]. The HusR regulon includes genes responsible for DNA biosynthesis or salvage in response to substrate availability, such as *xapA*, which is regulated by XapR in response to xanthine [42,43], and *nrdAB*, which is regulated by NrdR in response to ATP and dATP [44,45]. Additionally, this regulon includes *nrdAB* and *iraD*, which are regulated by DnaA to control replication initiation in response to the dATP/ATP ratio [46,47]. Therefore, because the HusR regulon contains genes regulated by transcription factors involved in the synthesis of DNA substrates and DNA replication, this supports the validity of the regulatory role of HusR in DNA-related functions in the stationary phase.

Deficiency of *husR* led to abnormal cell elongation and the appearance of anucleated cells in the presence of HU, an inhibitor of NDP reductase (NrdAB), resulting in dNTP depletion and inhibition of replication [Figure 4 and Figure 5, Table 2]. The effects of NDP reductase and dNTP depletion on *E. coli* were investigated in detail using experiments with the *nrdA* temperature-sensitive strain and HU, as described by the research group led by Guzmán [33,38,48]. They found that inactivating the enzymatic activity of *nrdA* or adding HU caused *E. coli* cells to elongate, led to the appearance of anucleate cells, and resulted in the generation of single-stranded DNA due to DNA damage [33,38]. Further analysis showed that the resolvasome RuvABC, recombination/repair protein RecA, and exodeoxyribonuclease RecBCD were involved in DNA damage repair [48]. *rusA* has been identified as a multicopy suppressor of UV sensitivity in the *ruvABC* deletion strain [49], functioning as an endonuclease for holliday junction resolvases [50]. It has also been reported that inhibition of NDP reductase by HU treatment induces hydroxyl radical-mediated cell death, which in response induces a set of genes managing genomic instability, including the *ruvAB* operon [51]. It is plausible that HusR affects HU sensitivity because it regulates genes involved in dNTP synthesis and DNA damage repair, as previously reported. The *rusA* phenotype was observed only upon plasmid overexpression, suggesting that its genomic expression levels were low [52]. To date, the regulation of *rusA* expression has been unclear, but this study revealed that *rusA* is expressed in the stationary phase upon activation with HusR.

HusR is also suggested to be involved in long-term survival, particularly under nitrogen-deficient conditions [Figure 6]. This is consistent with the finding that, in the stationary phase, the expression levels of *nrdAB* and *xapA* were dependent on HusR. [Figure 3]. It has been reported that the degradation of allantoin, a breakdown product of purine nucleotides, is necessary for *E. coli* long-term survival under nitrogen-starved conditions [53]. In recent years, we discovered that the sigma factor RpoN, a master regulator of nitrogen source metabolism, is required for the long-term survival of *E. coli* in soil [54]. HusR may be required for DNA maintenance during long-term survival because it regulates the Holliday junction resolvase RusA, the dNTP synthase NrdAB, and the purine salvage enzyme XapA.

## 5. Conclusions

We identified that the hitherto uncharacterized TF YnfL is a local regulator of substrate production for DNA synthesis and DNA repair. These findings contribute to understanding DNA maintenance and long-term survival through transcriptional regulation. Here, we propose renaming it HusR, as it determines HU sensitivity. Although the transcriptional level and phenotype of the HusR regulon, composed of several genes, have been elucidated, to further understand the role of HusR, it is necessary to determine its effects at the protein level and to identify the target gene(s) responsible for its phenotypes. HusR is a LysR-type transcription factor, and it has been suggested that it senses low-molecular-weight compounds as effector molecules. Future studies aimed at identifying this effector molecule, such as screening for metabolic intermediates known to accumulate under replication stress and targeted mutagenesis into the predicted ligand-binding domain within the HusR protein structure, are expected to reveal the physiological roles of HusR.

## Figures and Tables

**Table 1:** HusR-binding sites on the *E.coli* genome.

	Peak Position (bp)	Intensity	Function	Operon	Gene	D	Husr Site	D	Gene	Operon	Function	HusR Box CnGnnnAnnTnnnCnG
**1**	**393,934**	**78**			*yaiU*	>	***yaiV***	<	*ampH*			
**2**	**572,548**	**343**			*ybcO*	>		**>**	***rusA***	***rusA-ylcG***	**endodeoxyribonuclease** **(Holliday junction resolvase)**	**CGGGCGACGTTATCGC** **CAGGTAAGAATCCCTG**
**3**	**1,664,668**	**72**			*clcB*	<	***ynfK***	<	*dgsA*			
**4**	**1,667,732**	**191**	**predicted DNA-binding** **transcriptional regulator**	***husR*** **(*ynfL*)**	***husR*** **(*ynfL*)**	**<**		**>**	***ynfM***	***ynfM***	**putative transporter**	**CAGCCTATCCTTCCGG** **CCGGAAGGATAGGCTG**
**5**	**2,342,734**	**121**	**hypothetical protein**	***ypaB***	***ypaB***	**<**		**>**	***nrdA***	***nrdAB***	**ribonucleoside-diphosphate reductase**	**CAGACAAGGTGTCCGG** **CACTTTTTCCTTCCTG** **CCGGACACCTTGTCTG**
**6**	**2,523,166**	**113**	**xanthosine phosphorylase**	***xapA***	***xapA***	**<**		**>**	***yfeN***	***yfeN***	**putative outer membrane porin**	**GGGGAGAAATGTACGG** **TAGCGAGGTTGTCCCG**
**7**	**2,985,164**	**61**			*yqeG*	>		**>**	***yqeH***	***yqeH***	**putative transcription factor**	**CGCAAGATATCTTCTG**
**8**	**4,248,830**	**54**			*malM*	>		**>**	***yjbI***	***yjbI***	**function unknown conserved protein**	**CAGGCTACTTTTCCAG** **CTGGAAAAGTAGCCTG**
**9**	**4,554,598**	**88**			*ytiD*	>		**>**	***idlP***	***idlP-iraD***	***iraD* leader peptide**	**CAGAATATACTAGCGG** **CCGCTAGTATATTCTG**

HusR-binding sites indicated in green represent the HusR-binding sites inside spacer regions, whereas sites indicated in orange represent the HusR-binding sites inside ORFs. Column D indicates the direction of transcription. The genes filled with gray indicate the HusR-binding site is located downstream of the gene.

**Table 2:** Cell size and percentage of anucleate cells obtained after 24 h of addition of hydroxyurea.

	HU Conc.	Turbidity (O.D._600_)	Cell Size (µm)	Anucleates (%)
Wild-type strain	0 mM	2.10	1.9 ± 0.4	0.0
	50 mM	0.71	3.7 ± 1.7	25.1
	100 mM	0.09	5.9 ± 2.1	17.7
∆*husR* strain	0 mM	2.10	2.3 ± 0.4	0.0
	50 mM	1.13	5.4 ± 2.6	23.6
	100 mM	0.72	9.6 ± 9.1	73.7

## Data Availability

The original contributions presented in this study are included in the article/Supplementary Materials. Further inquiries can be directed to the corresponding authors.

## Supplementary Materials

The following supporting information can be downloaded at: https://www.mdpi.com/article/10.3390/microorganisms14010134/s1, Table S1: Primers used in this study. Figure S1. Histogram of cell length and the count after 24 h of addition of hydroxyurea (HU).

## Abbreviations

The following abbreviations are used in this manuscript:

*E. coli*	*Escherichia coli*
gSELEX	genomic SELEX
TF	transcription factor
HU	hydroxyurea
PAGE	polyacrylamide gel electrophoresis
NDP	nucleoside diphosphate
LB	Luria-Bertani
bp	base pair
ORF	open reading frame
PCR	polymerase chain reaction
RT-qPCR	Reverse Transcription-quantitative PCR

## References

[1] Wang B.B., Xu J.Z., Zhang F., Liu S., Liu J., Zhang W.G. (2022). Review of DNA repair enzymes in bacteria: With a major focus on AddAB and RecBCD. DNA Repair.

[2] Nudler E. (2023). Transcription-coupled global genomic repair in *E. coli*. Trends Biochem. Sci..

[3] Sharples G.J. (2001). The X philes: Structure-specific endonucleases that resolve Holliday junctions. Mol. Microbiol..

[4] Minsky A., Shimoni E., Frenkiel-Krispin D. (2002). Stress, order and survival. Nat. Rev. Mol. Cell Biol..

[5] Frenkiel-Krispin D., Minsky A. (2006). Nucleoid organization and the maintenance of DNA integrity in *E. coli*, *B. subtilis* and *D. radiodurans*. J. Struct. Biol..

[6] Mathews C.K. (2014). Deoxyribonucleotides as genetic and metabolic regulators. FASEB J..

[7] Wehrens M., Ershov D., Rozendaal R., Walker N., Schultz D., Kishony R., Levin P.A., Tans S.J. (2018). Size Laws and Division Ring Dynamics in Filamentous *Escherichia coli* cells. Curr. Biol..

[8] Blattner F.R., Plunkett G., Bloch C.A., Perna N.T., Burland V., Riley M., Collado-Vides J., Glasner J.D., Rode C.K., Mayhew G.F. (1997). The complete genome sequence of *Escherichia coli* K-12. Science.

[9] Hayashi K., Morooka N., Yamamoto Y., Fujita K., Isono K., Choi S., Ohtsubo E., Baba T., Wanner B.L., Mori H. (2006). Highly accurate genome sequences of *Escherichia coli* K-12 strains MG1655 and W3110. Mol. Syst. Biol..

[10] Ishihama A. (1997). Adaptation of gene expression in stationary phase bacteria. Curr. Opin. Genet. Dev..

[11] Ishihama A. (1999). Modulation of the nucleoid, the transcription apparatus, and the translation machinery in bacteria for stationary phase survival. Genes Cells.

[12] Wang X., Kim Y., Ma Q., Hong S.H., Pokusaeva K., Sturino J.M., Wood T.K. (2010). Cryptic prophages help bacteria cope with adverse environments. Nat. Commun..

[13] Soo V.W.C., Hanson-Manful P., Patrick W.M. (2011). Artificial gene amplification reveals an abundance of promiscuous resistance determinants in *Escherichia coli*. Proc. Natl. Acad. Sci. USA.

[14] Ishihama A., Kori A., Koshio E., Yamada K., Maeda H., Shimada T., Makinoshima H., Iwata A., Fujita N. (2014). Intracellular concentrations of 65 species of transcription factors with known regulatory functions in *Escherichia coli*. J. Bacteriol..

[15] Ishihama A., Shimada T., Yamazaki Y. (2016). Transcription profile of *Escherichia coli*: Genomic SELEX search for regulatory targets of transcription factors. Nucleic Acids Res..

[16] Shimada T., Ogasawara H., Ishihama A. (2018). Single-target regulators form a minor group of transcription factors in *Escherichia coli* K-12. Nucleic Acids Res..

[17] Shimada T., Ogasawara H., Ishihama A. (2018). Genomic SELEX screening of regulatory targets of *Escherichia coli* transcription factors. Bacterial Chromatin.

[18] Shimada T., Ogasawara H., Kobayashi I., Ishihama A. (2024). Genomic SELEX screening of regulatory targets of transcription factors. Bacterial Chromatin.

[19] Ishihama A. (2010). Prokaryotic genome regulation: Multifactor promoters, multitarget regulators and hierarchic networks. FEMS Microbiol. Rev..

[20] Ishihama A., Shimada T. (2021). Hierarchy of transcription factor network in *Escherichia coli* K-12: H-NS-mediated silencing and anti-silencing by global regulators. FEMS Microbiol. Rev..

[21] Jishage M., Ishihama A. (1997). Variation in RNA polymerase sigma subunit composition within different stocks of *Escherichia coli* W3110. J. Bacteriol..

[22] Datsenko K.A., Wanner B.L. (2000). One-step inactivation of chromosomal genes in *Escherichia coli* K-12 using PCR products. Proc. Natl. Acad. Sci. USA.

[23] Baba T., Ara T., Hasegawa M., Takai Y., Okumura Y., Baba M., Datsenko K.A., Tomita M., Wanner B.L., Mori H. (2006). Construction of *Escherichia coli* K-12 in-frame, single-gene knockout mutants: The Keio collection. Mol. Syst. Biol..

[24] Bordat A., Houvenaghel M.C., German-Retana S. (2015). Gibson assembly: An easy way to clone potyviral full-length infectious cDNA clones expressing an ectopic VPg. Virol. J..

[25] Shimada T., Fujita N., Maeda M., Ishihama A. (2005). Systematic search for the Cra-binding promoters using genomic SELEX system. Genes Cells.

[26] Shimada T., Ishihama A., Busby S.J.W., Grainger D.C. (2008). The *Escherichia coli* RutR transcription factor binds at targets within genes as well as intergenic regions. Nucleic Acids Res..

[27] Shimada T., Tanaka K., Ishihama A. (2016). Transcription factor DecR (YbaO) controls detoxification of L-cysteine in *Escherichia coli*. Microbiology.

[28] Anzai T., Imamura S., Ishihama A., Shimada T. (2020). Expanded roles of pyruvate-sensing PdhR in transcription regulation of the *Escherichia coli* K-12 genome: Fatty acid catabolism and cell motility. Microb. Genom..

[29] Gutnick D., Calvo J.M., Klopotowski T., Ames B.N. (1969). Compounds which serve as the source of carbon or nitrogen for *Salmonella typhimurium* LT-2. J. Bacteriol..

[30] Shimada T., Bridier A., Briandet R., Ishihama A. (2011). Novel roles of LeuO in transcription regulation of *E. coli* genome: Antagonistic interplay with the universal silencer H-NS. Mol. Microbiol..

[31] Ishihama A. (2012). Prokaryotic genome regulation: A revolutionary paradigm. Proc. Jpn. Acad. Ser. B Phys. Biol. Sci..

[32] Bailey T.L., Boden M., Buske F.A., Frith M., Grant C.E., Clementi L., Ren J., Li W.W., Noble W.S. (2009). MEME SUITE: Tools for motif discovery and searching. Nucleic Acids Res..

[33] Guarino E., Jiménez-Sánchez A., Guzmán E.C. (2007). Defective ribonucleoside diphosphate reductase impairs replication fork progression in *Escherichia coli*. J. Bacteriol..

[34] Merrikh H., Ferrazzoli A.E., Bougdour A., Olivier-Mason A., Lovett S.T. (2009). A DNA damage response in *Escherichia coli* involving the alternative sigma factor, RpoS. Proc. Natl. Acad. Sci. USA.

[35] Merrikh H., Ferrazzoli A.E., Lovett S.T. (2009). Growth phase and (p)ppGpp control of IraD, a regulator of RpoS stability, in *Escherichia coli*. J. Bacteriol..

[36] Nishino K., Yamaguchi A. (2001). Analysis of a complete library of putative drug transporter genes in *Escherichia coli*. J. Bacteriol..

[37] Britton R.A., Lupski J.R. (1997). Isolation and characterization of suppressors of two *Escherichia coli dnaG* mutations, *dnaG2903* and *parB*. Genetics.

[38] Riola J., Guarino E., Guzmán E.C., Jiménez-Sánchez A. (2007). Differences in the degree of inhibition of NDP reductase by chemical inactivation and by the thermosensitive mutation *nrdA101* in *Escherichia coli* suggest an effect on chromosome segregation. Cell Mol. Biol. Lett..

[39] Ishihama A. (2018). Building a complete image of genome regulation in the model organism *Escherichia coli*. J. Gen. Appl. Microbiol..

[40] Gao Y., Lim H.G., Verkler H., Szubin R., Quach D., Rodionova I., Chen K., Yurkovich J.T., Cho B.K., Palsson B.O. (2021). Unraveling the functions of uncharacterized transcription factors in *Escherichia coli* using ChIP-exo. Nucleic Acids Res..

[41] Hemm M.R., Paul B.J., Schneider T.D., Storz G., Rudd K.E. (2008). Small membrane proteins found by comparative genomics and ribosome binding site models. Mol. Microbiol..

[42] Seeger C., Poulsen C., Dandanell G. (1995). Identification and characterization of genes (*xapA*, *xapB*, and *xapR*) involved in xanthosine catabolism in *Escherichia coli*. J. Bacteriol..

[43] Jørgensen C., Dandanell G. (1999). Isolation and characterization of mutations in the *Escherichia coli* regulatory protein XapR. J. Bacteriol..

[44] McKethan B.L., Spiro S. (2013). Cooperative and allosterically controlled nucleotide binding regulates the DNA binding activity of NrdR. Mol. Microbiol..

[45] Rozman Grinberg I., Bimaï O., Shahid S., Philipp L., Martínez-Carranza M., Banerjee I., Lundin D., Stenmark P., Sjöberg B.M., Logan D.T. (2025). Bacterial transcriptional repressor NrdR—A flexible multifactorial nucleotide sensor. FEBS J..

[46] Sass T.H., Ferrazzoli A.E., Lovett S.T. (2022). DnaA and SspA regulation of the *iraD* gene of *Escherichia coli*: An alternative DNA damage response independent of LexA/RecA. Genetics.

[47] Stringer A.M., Fitzgerald D.M., Wade J.T. (2024). Mapping the *Escherichia coli* DnaA-binding landscape reveals a preference for binding pairs of closely spaced DNA sites. Microbiology.

[48] Salguero I., Guarino E., Guzmán E.C. (2011). RecA-dependent replication in the *nrdA101*(Ts) mutant of *Escherichia coli* under restrictive conditions. J. Bacteriol..

[49] Mandal T.N., Mahdi A.A., Sharples G.J., Lloyd R.G. (1993). Resolution of Holliday intermediates in recombination and DNA repair: Indirect suppression of *ruvA*, *ruvB*, and *ruvC* mutations. J. Bacteriol..

[50] Sharples G.J., Chan S.N., Mahdi A.A., Whitby M.C., Lloyd R.G. (1994). Processing of intermediates in recombination and DNA repair: Identification of a new endonuclease that specifically cleaves Holliday junctions. EMBO J..

[51] Davies B.W., Kohanski M.A., Simmons L.A., Winkler J.A., Collins J.J., Walker G.C. (2009). Hydroxyurea induces hydroxyl radical-mediated cell death in *Escherichia coli*. Mol. Cell.

[52] Mahdi A.A., Sharples G.J., Mandal T.N., Lloyd R.G. (1996). Holliday junction resolvases encoded by homologous *rusA* genes in *Escherichia coli* K-12 and phage 82. J. Mol. Biol..

[53] Switzer A., Burchell L., McQuail J., Wigneshweraraj S. (2020). The adaptive response to long-term nitrogen starvation in *Escherichia coli* requires the breakdown of allantoin. J. Bacteriol..

[54] Nakamoto S., Kobayashi I., Watanabe K., Kikuta T., Imamura S., Shimada T. (2025). Identification of a comprehensive set of transcriptional regulators involved in the long-term survivability of *Escherichia coli* in soil. Sci. Rep..

