# Supplementary material for: Transcription Factor HusR (YnfL) Is a Novel Regulator for Hydroxyurea Sensitivity in Escherichia coli K-12
Source: Microorganisms. 2026 Jan 7;14(1):134. doi: 10.3390/microorganisms14010134 (PMC12844172; doi:10.3390/microorganisms14010134)
Supplement: Supplementary file 1 [file microorganisms-14-00134-s001.zip › microorganisms-4039986-supplementary.pdf]

Table S1. Primers used in this study.

(A) For gel shift assay.

name	sequence
-/rusA-F	GAAACGTCTGTACTGGCACATATCC
-/rusA-R	-/rusA-R GGCGACGTTATCGCGGTATG
husR/ynfM-F	GCTGCCGTCAGCAATACAC
husR/ynfM-R	GCTTTGCTTGTCAGTGTCGC
ypaB/nrdA-F	CCCGGTGCAATGGCTATTTTG
ypaB/nrdA-R	ypaB/nrdA-R CCGGGAAAGTCAATGGGAAG
xapA/yfeN-F	TCCTTTTTCTGTAGGGTGGAATCTAAC
xapA/yfeN-R	xapA/yfeN-R GGTAGTCCGGCTGGTCC
-/yqeH-F	TCGTGGGTATCCTGTGCGTATC
-/yqeH-R	CTGATTTAATCTTGCATGGTTGAGAGC
-/yjbI-F	CTTGAAATCGAAACACTACCATTTGATGAG
-/yjbI-R	GTCCTCTTACTCGTCATTAATAATCGTAACAATAC
-/idlP-F	GGGTAGGAAACACTCTAAAGTATCAAAAAAC
-/idlP-R	CCTTACTTGCCTATGAATATCTACTGAC
lacUV5-F	CAGCTGGCACGACAGGTTTC
lacUV5-R	AGCTGTTTCCTGTGTGAAATTG

(B) For RT-qPCR analysis.

name	sequence
rusA-F	GCAAAAAGCCGCTTTTGACG
rusA-R	AACAGGCATCTTCACAACGC
ylcG-F	TGACGCGATGATCATTTGCG
ylcG-R	AACCATCAGAGCAGCAATGC
husR-F	TGAACACTCGCGAGCAAATC
husR-R	ACGATGACTGCGTGTTCAAG
ynfM-F	ATTATGCGCGCCTTGATTGG
ynfM-R	TCATGCCGCCAATTGAGTTG
ypaB-F	ATGACCCTCTTGCAAGTGC
ypaB-R	TGACTTTCCCGGACACCTTG
nrdA-F	AATCAAAACGCACGGTCTGC
nrdA-R	TTCGACGCTTTGATGCTGAC
nrdB-F	ACTGTATCTCTGCCTGATGAGC
nrdB-R	TGCGAAGGAACAAGCAAAGC
xapA-F	TATGGCGGAAGGTTTAAGCG
xapA-R	GCAATTTTGCGCAGAAAGCC
yfeN-F	AGGCGGTTTTGCAGATATCG
yfeN-R	GCGCCACCTTCAAATTCAAG
yqeH-F	TCACCGCGTTTGCAGTATTG
yqeH-R	CCGCTTAATCTCGCATTGTGG
yjbI-F	TAAGCTCCCAGCGCTTACAT
yjbI-R	CGCAGTAAAAATGGGGAAAA
idlP-F	GGAAAATGAGCATCAATACAGTGG
idlP-R	TCATTTTGCGCACTCCTGAC
iraD-F	TTTCATGAGCCACGGATTGC
iraD-R	AACGTTAGCGGTTCATCGTC

gfpuv-F	ATGAGTAAAGGAGAAGAACTTTTCACTGG
gfpuv-R	CCACTGACAGAAAATTTGTGCCC

(C) For construction of husR promoter fused GFPuv plasmid.

name	sequence
GFPuv-F	ATGAGTAAAGGAGAAGAACTTTTCACTGG
GFPuv-R	TCACTGCCCGCTTTCCAG
PhusR-F	GAAAGCGGGCAGTGACGCGCATAATCAAAATGCCGTG
PhusR-R	CTCCTTTACTCATCAGATGACGAAGTTCAATATTCATATTTAA

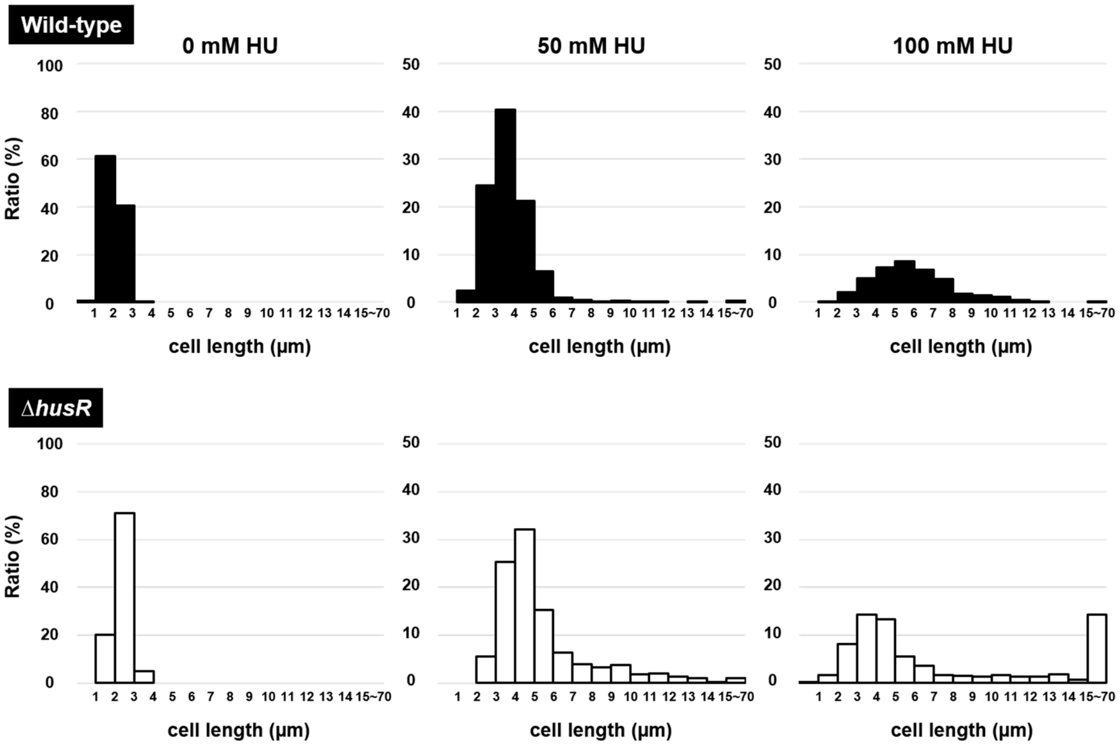

Figure S1. Histogram of cell length and the count after 24h of addition of hydroxyurea (HU). Histograms show the cell length and the count of *E. coli* wild-type BW25113 and Δ*husR* JW1587 strains after 24h of addition of HU. The histograms are normalized to the total count of measured cells. The x-axis represents cell length, with cells longer than 15 μm grouped into bins labeled 15–70, and the y-axis represents the fraction of the cell population.
